# Non-Glycemic Clinical Data for Type 2 Diabetes Detection in Mexican Adults: A Comparative Analysis of Atherogenic Indices, Statistical Transformations, and Machine Learning Algorithms

**DOI:** 10.3390/diagnostics16010053

**Published:** 2025-12-23

**Authors:** Martin Hazael Guerrero-Flores, Valeria Maeda-Gutiérrez, Carlos E. Galván-Tejada, Jorge I. Galván-Tejada, Miguel Cruz, Luis Alberto Flores-Chaires, Karina Trejo-Vázquez, Rafael Magallanes-Quintanar, Javier Saldívar

**Affiliations:** 1 Unidad Académica de Ingeniería Eléctrica, Universidad Autónoma de Zacatecas, Jardín Juarez 147, Centro, Zacatecas 98000, Mexico; hazaelgf@uaz.edu.mx (M.H.G.-F.); ericgalvan@uaz.edu.mx (C.E.G.-T.); luischaires@uaz.edu.mx (L.A.F.-C.); ktrejov@uaz.edu.mx (K.T.-V.); tiquis@uaz.edu.mx (R.M.-Q.); javier.saldivarp@uaz.edu.mx (J.S.); 2 Unidad de Investigación Médica en Bioquímica, Hospital de Especialidades, Centro Médico Nacional Siglo XXI, Instituto Mexicano del Seguro Social, Av. Cuauhtémoc 330, Col. Doctores, Del. Cuauhtémoc, Mexico City 06720, Mexico; mcruz@yahoo.com

**Keywords:** type 2 diabetes, non-glycemic biomarkers, lipid profile, atherogenic indices, machine learning

## Abstract

**Background:** Type 2 diabetes (T2D) is a growing public health problem in Mexico. Lipid profile alterations have been shown to appear years before changes in glycemic biomarkers, and some of the latter are limited in availability, especially in underserved settings. Therefore, anthropometric variables and lipids represent relevant early indicators for the early detection of the disease. This study evaluates the capacity of non-glycemic clinical data—including lipid profile and anthropometric indicators—to detect T2D using machine learning, and compares the performance of different feature engineering approaches. **Methods:** Using more than a thousand clinical records of Mexican adults, three experiments were developed: (1) a distribution and normality analysis to characterize the variability of lipid variables; (2) an evaluation of the predictive power of multiple atherogenic indices (Castelli I, Castelli II, TG/HDL, and AIP); and (3) the implementation of statistical transformations (logarithmic, quare-root, and Z-standardization) to stabilize variance and improve feature quality. Logistic regression, SVM-RBF, random forest, and XGBoost models were trained on each feature set and evaluated using accuracy, sensitivity, specificity, F1-score, and area under the ROC curve. **Results:** The AIP index showed the greatest discriminatory power among the atherogenic indices, while normality-based transformations improved the performance of distribution-sensitive models, such as SVM. In the final experiment, the SVM-RBF and XGBoost models achieved AUC values greater than 0.90, demonstrating the feasibility of a diagnostic approach based exclusively on non-glycemic data. **Conclusions:** The findings indicate that the transformed lipid profile and anthropometric variables can constitute a solid and accessible alternative for the early detection of T2D in clinical and public health contexts, offering a robust methodological framework for future predictive applications in the absence of traditional glycemic biomarkers.

## 1. Introduction

Type 2 diabetes (T2D) is one of the leading global causes of morbidity and mortality, with an estimated 537 million adults affected in 2021 and projections suggesting that this number will reach 643 million by 2030, according to the International Diabetes Federation (IDF) [1]. In parallel, cardiovascular diseases (CVDs) remain the primary cause of death worldwide and share multiple metabolic risk factors with T2D, including dyslipidemia and insulin resistance [2].

In Mexico, the burden of T2D is particularly alarming. Recent analyses of nationally representative surveys show that the prevalence of diabetes increased between 2016 and 2022, reaching 15.7% in adults, with marked sociodemographic inequalities [3]. ENSANUT 2022 additionally reported that 12.3% of adults have diabetes, while 18.2% present prediabetes, reinforcing an upward trend observed for more than two decades [4]. Mortality associated with T2D has also grown, becoming one of the leading causes of death at the municipal level across the country [5]. These indicators highlight the urgent need for more accessible and scalable strategies for early detection.

Traditional diagnostic markers such as fasting plasma glucose, the oral glucose tolerance test (OGTT), and HbA1c remain the clinical standard for the diagnosis of type 2 diabetes [6,7]. These tests are widely used in clinical practice and rely on standardized laboratory procedures, including fasting conditions and confirmatory measurements when required. However, accumulating evidence from metabolic studies indicates that alterations in lipid metabolism and other metabolic pathways may occur several years before glycemic abnormalities become clinically detectable, suggesting that relevant pathophysiological changes precede the onset of overt dysglycemia [8]. In particular, atherogenic dyslipidemia—characterized by elevated triglycerides, reduced HDL cholesterol, and increased small dense LDL particles—has been shown to occur early in the cardiometabolic continuum and is associated with an increased risk of progression toward type 2 diabetes [9,10]. These early lipid abnormalities suggest that lipid-derived markers may serve as valuable non-glycemic biomarkers for anticipatory risk identification [11].

Among lipid-based indicators, atherogenic indices such as the Castelli ratios, triglyceride-to-HDL ratio (TG/HDL), and the Atherogenic Index of Plasma (AIP) have demonstrated strong associations with insulin resistance, prediabetes, and cardiometabolic risk [2,12]. Recent cohort studies show that AIP exhibits nonlinear and predictive relationships with both prediabetes and T2D incidence [13,14]. In the Mexican population, lipid alterations are also frequently observed in individuals with insulin resistance and undiagnosed diabetes, further supporting their diagnostic relevance [15].

The potential of these non-glycemic variables can be further enhanced through machine learning (ML) techniques, which have shown promising performance in diabetes prediction using clinical, paraclinical, and lifestyle data [16]. Previous work demonstrated that AIP, was a lipid-derived predictor for distinguishing diabetic from non-diabetic adults [13].

Building on this foundation, the present study extends the analytical framework by incorporating lipid variable transformations guided by formal normality assessments and comprehensive feature engineering pipelines, with the specific objective of enhancing the discrimination between adults with previously undiagnosed type 2 diabetes and those without diabetes at the time of evaluation.

Accordingly, this research aims to systematically assess the diagnostic performance of non-glycemic clinical data—including atherogenic indices, statistically transformed lipid traits, and anthropometric variables—for the identification of type 2 diabetes in Mexican adults who have not been previously diagnosed, using multiple machine learning models.

## 2. Materials and Methods

**Figure 1:**
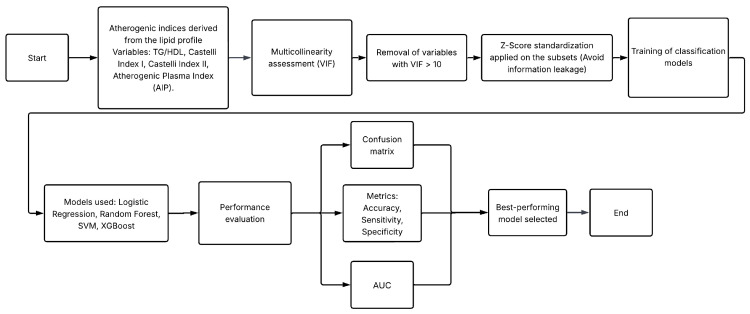
Methodological workflow followed in Experiment 1.

**Figure 2:**
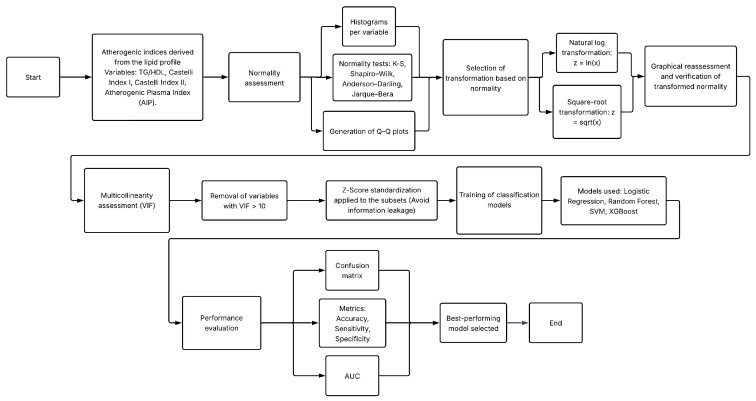
Methodological workflow followed in Experiment 2, including normality assessment and variable transformations.

This study was structured into two main experiments designed to evaluate the diagnostic potential of non-glycemic clinical variables—specifically lipid profile components, derived atherogenic indices, and anthropometric measurements—for the identification of T2D. Both experiments used the same clinical database but differed in the preprocessing strategies applied to the lipid variables. Figure 1 and Figure 2 summarize the methodological workflow followed in Experiment 1 and Experiment 2, respectively.

- Experiment 1 assessed the predictive value of atherogenic indices and anthropometric variables after removing glycemic biomarkers and controlling for multicollinearity. The complete workflow is in Figure 1.
- Experiment 2 extended this framework by incorporating formal normality testing and applying statistical transformations (logarithmic and square-root) to improve distributional properties of lipid variables prior to model training. The processing pipeline is in Figure 2.

The following subsections describe the dataset, preprocessing procedures, exploratory analyses, normality assessments, feature engineering steps, and the experimental design adopted for both experiments.

### 2.1. Database Description

The clinical dataset analyzed in this study was obtained from the “Unidad de Investigación Médica en Bioquímica, Centro Médico Nacional Siglo XXI, Instituto Mexicano del Seguro Social (IMSS)”. All participants provided written informed consent, and the study protocol complied with the principles of the Declaration of Helsinki and was approved by the IMSS Ethics Committee (Approval No. R-2011-785-018). The study population corresponds to a previously established clinical cohort of adult Mexican individuals aged between 35 and 65 years at the time of evaluation. Participants classified as T2D cases were identified according to the American Diabetes Association (ADA) criteria, including fasting plasma glucose levels equal to or greater than 126 mg/dL. Both male and female participants were included, provided that they were active IMSS beneficiaries at the time of enrollment and agreed to participate in the study. To avoid potential familial clustering effects, individuals with family members enrolled in the study were excluded.

The control group consisted of adult individuals within the same age range who did not meet the criteria for metabolic syndrome, based on the Adult Treatment Panel III (ATP III) guidelines. Controls exhibited fasting glucose levels below 100 mg/dL and post-load glucose levels below 140 mg/dL two hours after the ingestion of 75 g of glucose. As in the case group, both sexes were included, and all participants provided informed consent and met institutional eligibility requirements.

Exclusion criteria for both groups included individuals with temporary or seasonal insurance coverage, those without permanent residence or who could not be reliably contacted by telephone, and women in climacteric stage.

The dataset comprises laboratory-confirmed fasting measurements and electronic clinical records from a cohort of adult Mexican individuals aged between 35 and 65 years. The final dataset includes a total of 1019 subjects, Of these, 517 were men and 502 were women. According to established clinical diagnostic criteria, 520 people were classified as having T2D, while 499 were categorized as non-diabetic.

Each record contains standardized anthropometric measurements (weight, height, body mass index, waist and hip circumference, and waist-to-hip ratio), hemodynamic parameters (systolic and diastolic blood pressure), and a detailed fasting lipid profile that includes total cholesterol, LDL, HDL, and triglycerides. Additionally, the database includes biochemical glycemic markers such as fasting glucose (mg/dL), glucose in mmol/L, insulin, and the Homeostatic Model Assessment of Insulin Resistance (HOMA-IR).

All laboratory measurements were obtained under standardized clinical protocols and validated for completeness. Records with missing or inconsistent biochemical measurements were discarded to ensure analytical validity. A complete list of the variables included in the dataset is presented in Table 1.

### 2.2. Preprocessing

This section describes the data preprocessing procedures applied before model development. The workflow included the primary exclusion of glycemic variables, as well as the calculation and integration of atherogenic indices derived from lipid measurements to enrich the predictive information of the dataset. An exploratory assessment of feature distributions and correlations was then performed to identify redundancy and multicollinearity among variables, along with a normality analysis to determine the transformations required to stabilize variance and reduce skewness.

Subsequently, in Experiment 1, all continuous non-glycemic clinical features were standardized using Z-score normalization to ensure numerical consistency across heterogeneous units, a procedure widely recommended in clinical predictive modeling and machine learning. In contrast, Experiment 2 did not apply Z-score standardization after the transformations, as these statistical corrections inherently modified the scale and distribution of the lipid variables.

These preprocessing steps ensured proper formatting of the data, improved the suitability of the features for modeling, and enabled a consistent and comparable evaluation of the machine learning algorithms across both experiments.

#### 2.2.1. Exclusion of Glycemic and Non-Informative Variables

Consistent with the non-glycemic diagnostic objective of this study, all variables directly associated with glucose metabolism were removed prior to model development. Although these features were available in the original clinical dataset, their inclusion would contradict the aim of evaluating lipid-derived and anthropometric predictors of type 2 diabetes (T2D) independent of traditional glycemic biomarkers. Emerging evidence indicates that metabolic disturbances—particularly dyslipidemia—may precede detectable alterations in glycemia by several years [2,17]. The excluded glycemic variables were: Glucose, MmolGluc, Insulin, and HOMA_IR.

Additionally, the ID variable was excluded, as it functions solely as a patient identifier and does not contribute any clinical, biochemical, or predictive information to the modeling process.

#### 2.2.2. Calculation and Incorporation of Atherogenic Indices

To enhance the predictive capacity of the clinical dataset, several atherogenic indices were computed from the fasting lipid profile. These indices are widely recognized as indirect markers of cardiovascular and metabolic risk and have been associated with early metabolic impairment and future T2D development [12,15]. All indices were derived using total cholesterol, HDL cholesterol, LDL cholesterol, and triglycerides, following standard formulations reported in clinical research.

The atherogenic indices calculated in this study were:

(1) $$\mathrm{Castelli}\;\mathrm{Index}\;I=\frac{\mathrm{Total}\;\mathrm{Cholesterol}}{\mathrm{HDL}\;\mathrm{Cholesterol}}$$

(2) $$\mathrm{Castelli}\;\mathrm{Index}\;\mathrm{II}=\frac{\mathrm{LDL}\;\mathrm{Cholesterol}}{\mathrm{HDL}\;\mathrm{Cholesterol}}$$

(3) $$\mathrm{TG}/\mathrm{HDL}\;\mathrm{Ratio}=\frac{\mathrm{Triglycerides}}{\mathrm{HDL}\;\mathrm{Cholesterol}}$$

(4) $$\mathrm{AIP}=\log_{10}\left(\frac{\mathrm{Triglycerides}}{\mathrm{HDL}\;\mathrm{Cholesterol}}\right)$$

These derived variables were incorporated into the dataset under the names IC1, IC2, TG_HDL, and AIP, respectively, supporting their evaluation as non-glycemic predictors in type 2 diabetes classification.

#### 2.2.3. Exploratory Analysis: Histograms and Correlation Assessment

Before developing the predictive models, an exploratory analysis was performed to characterize the statistical behavior of all non-glycemic clinical variables. First, histograms were generated for each continuous feature to identify skewness, kurtosis, and potential outliers, following standard recommendations for descriptive analysis in health sciences [18]. This visualization highlighted, for instance, the strong right skewness of triglycerides and the TG/HDL ratio, a pattern frequently reported in populations with atherogenic dyslipidemia [2,15].

Additionally, a Pearson correlation matrix was computed to explore linear associations among continuous clinical and lipid-related variables. This approach is commonly used to analyze cardiometabolic relationships in clinical research [17]. The correlation matrix revealed strong associations among total cholesterol, LDL, and the Castelli indices, suggesting potential redundancy that was later examined through multicollinearity analysis.

#### 2.2.4. Multicollinearity Assessment

Multicollinearity among lipid components and derived atherogenic indices was assessed using the Variance Inflation Factor (VIF), a widely accepted criterion for identifying redundant predictors in biomedical datasets [19]. Features with VIF values greater than 10 were considered highly collinear. This analysis led to the exclusion of IC1, IC2, and TG/HDL in Experiment 1, as they exhibited substantial linear dependence with total cholesterol, LDL, and HDL. AIP was retained because of its lower VIF and its ability to capture meaningful metabolic information through the logarithmic transformation of TG/HDL [12].

#### 2.2.5. Formal Normality Assessment and QQ Plots (Experiment 2 Only)

Some tests are performed exclusively in experiment 2, such as to further assess deviations from normality, quantile–quantile (QQ) plots were produced for all continuous variables. These graphical diagnostics compare empirical quantiles to those of a theoretical normal distribution and are widely used in clinical statistical practice [20].

To determine the appropriate transformations to stabilize variance and reduce skewness before model training, normality tests were performed. Following established methodological guidelines [21,22], four normality tests were applied to each continuous feature: Kolmogorov–Smirnov, Shapiro–Wilk, Anderson–Darling, and Jarque–Bera. In all tests, the null hypothesis assumed a normal distribution; p-values less than 0.05 were interpreted as significant deviations from normality.

Results indicated pronounced deviations from normality in several lipid variables, particularly triglycerides and TG/HDL, which exhibited heavy right-tailed distributions. These findings motivated the use of transformation techniques to correct distributional irregularities in Experiment 2.

#### 2.2.6. Statistical Transformations Guided by Normality (Experiment 2 Only)

Based on the normality test results, a variable-specific transformation strategy was implemented to correct skewness and stabilize variance, following the recommendations of Osborne [22]. The following transformations were applied:

- Variables with strong right skewness (e.g., triglycerides, TG/HDL) were transformed using the natural logarithm.
- Variables with moderate skewness were transformed using square-root transformations.
- Variables exhibiting approximately symmetric distributions were left untransformed.

#### 2.2.7. Definition of Feature Sets and Experimental Design

Two independent experiments were conducted to evaluate the predictive potential of non-glycemic clinical variables for type 2 diabetes classification.

##### Experiment 1: Baseline Model with Atherogenic Indices

Experiment 1 included anthropometric variables, blood pressure measurements, and atherogenic indices (IC1, IC2, TG/HDL, and AIP). After performing the VIF analysis, only non-collinear predictors were retained, with AIP emerging as the main lipid-derived indicator due to its low VIF and its established diagnostic relevance in metabolic disorders [12].

In this experiment, all continuous variables were standardized using Z-score normalization prior to splitting the data into training and testing subsets (70–30%). This preprocessing step ensured consistent numerical scaling across the dataset and improved the behavior of machine learning algorithms sensitive to feature magnitude.

##### Experiment 2: Extended Model with Normality-Guided Transformations

Experiment 2 expanded the feature set by incorporating statistically transformed lipid variables, derived from the normality assessment. Logarithmic and square-root transformations were applied according to the degree of skewness observed in each lipid component. Because these transformations inherently altered the scale and dispersion of the variables, no Z-score normalization was applied in this experiment.

A second VIF analysis was performed after the transformations to ensure that no residual multicollinearity persisted. The resulting transformed feature set was then used for model training and evaluation, enabling a direct comparison with Experiment 1 and isolating the contribution of distributional corrections.

### 2.3. Machine Learning Algorithms

Four supervised machine learning algorithms were employed to evaluate the predictive capacity of non-glycemic clinical variables for distinguishing between diabetic and non-diabetic individuals. These models were selected to represent a diverse set of analytical paradigms, including linear classifiers, kernel-based methods, ensemble techniques, and gradient-boosted decision trees. This diversity enables a robust assessment of how different modeling approaches respond to the feature transformations and preprocessing strategies implemented in each experiment.

#### 2.3.1. Logistic Regression (LR)

Logistic Regression is a widely used linear classifier in clinical prediction tasks because of its interpretability and strong statistical foundations. It models the log-odds of class membership as a linear combination of the predictors, making it suitable as a baseline model in biomedical classification settings [23]. In this study, a binary logistic regression model was fitted using a binomial family with a logit link function, and model parameters were estimated via maximum likelihood. All selected non-glycemic features were included simultaneously in the model without applying regularization. Although LR assumes linearity in the logit and can be sensitive to multicollinearity and non-linear effects, it provides an essential point of comparison for evaluating the contribution of transformed and non-transformed lipid variables.

#### 2.3.2. Support Vector Machine with Radial Basis Function Kernel (SVM-RBF)

Support Vector Machines are powerful margin-based classifiers capable of learning complex decision boundaries. The use of a Radial Basis Function (RBF) kernel enables the mapping of input features into a higher-dimensional space, allowing the algorithm to model nonlinear relationships between predictors and outcomes [24]. SVM-RBF is particularly sensitive to feature scaling, which makes it informative for assessing the impact of transformation strategies applied in Experiment 2. SVMs have demonstrated strong performance in several biomedical predictive tasks, including diabetes screening.

#### 2.3.3. Random Forest (RF)

Support Vector Machines are powerful margin-based classifiers capable of learning complex decision boundaries. The use of a Radial Basis Function (RBF) kernel enables the mapping of input features into a higher-dimensional space, allowing the algorithm to model nonlinear relationships between predictors and outcomes [24]. In this study, an SVM classifier with an RBF kernel was trained using a cost parameter C=1, while the kernel width parameter γ was automatically set as the inverse of the number of input features. Probability estimates were enabled via Platt scaling to allow probabilistic output. SVM-RBF is particularly sensitive to feature scaling, which makes it informative for assessing the impact of transformation strategies applied in Experiment 2. SVMs have demonstrated strong performance in several biomedical predictive tasks, including diabetes screening.

#### 2.3.4. Extreme Gradient Boosting (XGBoost)

XGBoost is a highly optimized implementation of gradient-boosted decision trees that uses additive model building, regularization, and second-order gradient information to achieve superior predictive performance [25]. In this study, XGBoost was implemented using a tree-based booster (gbtree) with a binary logistic objective function. The model was trained for 100 boosting rounds (nrounds = 100), while the remaining hyperparameters were kept at their default values, including a learning rate (η) of 0.3 and a maximum tree depth of 6. No additional subsampling or explicit regularization strategies were applied. XGBoost is recognized for its efficiency, handling of missing values, and ability to model complex nonlinear patterns, often outperforming other tree-based ensemble methods in structured biomedical datasets. Its strong performance makes it a key model for evaluating the diagnostic potential of transformed lipid variables in Experiment 2.

Together, these four algorithms provide complementary perspectives on the classification task, enabling a comprehensive assessment of how feature engineering, multicollinearity control, and statistical transformations influence predictive performance across different modeling paradigms.

### 2.4. Evaluation Metrics

The evaluation of the classification models in both experiments was carried out using standard performance metrics derived from the confusion matrix. This matrix summarizes the relationship between the predicted and actual class labels and provides the foundational components for all subsequent diagnostic measures. Table 2 illustrates the general structure of the confusion matrix, which distinguishes between true positives (TP), true negatives (TN), false positives (FP), and false negatives (FN).

Based on these four quantities, several diagnostic metrics were computed to capture different aspects of model performance [26,27]:

- Accuracy evaluates the overall proportion of correctly classified samples:
  $$\mathrm{Accuracy}=\frac{TP+TN}{TP+TN+FP+FN}.$$
- Sensitivity (Recall) measures the model’s ability to correctly identify diabetic individuals:
  $$\mathrm{Sensitivity}=\frac{TP}{TP+FN}.$$
- Specificity quantifies the ability to correctly identify non-diabetic individuals:
  $$\mathrm{Specificity}=\frac{TN}{TN+FP}.$$
- Precision evaluates the proportion of predicted diabetic cases that are truly diabetic:
  $$\mathrm{Precision}=\frac{TP}{TP+FP}.$$
- F1-score is the harmonic mean of precision and recall, providing a balanced measure of the model’s performance when false positives and false negatives carry similar clinical relevance:
  $$F1\text{-}\mathrm{score}=2\cdot \frac{\mathrm{Precision}\cdot \mathrm{Recall}}{\mathrm{Precision}+\mathrm{Recall}}.$$

In addition to confusion-matrix-based metrics, the discriminative ability of each model was further assessed using the Receiver Operating Characteristic (ROC) curve, which plots the true positive rate against the false positive rate across varying decision thresholds. The Area Under the ROC Curve (AUC) provides a threshold-independent measure of separability between diabetic and non-diabetic individuals [28]. AUC values range from 0.5 (no discriminative ability) to 1.0 (perfect classification). Numerical integration using the trapezoidal rule was employed to estimate the AUC based on the ROC points obtained from the predicted probabilities.

Together, these metrics offer a comprehensive evaluation of model performance, capturing overall accuracy, class-specific performance, error balances, and threshold-independent discriminative ability. This multidimensional evaluation is fundamental when assessing predictive models intended for clinical decision support.

## 3. Results

This section presents the results obtained from the two experiments designed to evaluate the predictive capability of non-glycemic clinical features for T2D classification. The analyses include descriptive visual assessments of feature distributions, multicollinearity diagnostics, the impact of statistical transformations, and the performance of machine learning models across both experiments.

Experiment 1 provides a baseline evaluation using anthropometric variables, blood pressure measurements, and atherogenic indices, following Z-score standardization of continuous features. Histogram visualizations and correlation matrices were used to characterize the distributional behavior and linear relationships among clinical variables, while a Variance Inflation Factor (VIF) analysis guided the removal of redundant lipid predictors before model training.

Experiment 2 extends this framework by incorporating normality-driven transformations applied to the lipid profile components. To assess distributional improvements, histograms and quantile–quantile (QQ) plots were generated both before and after applying logarithmic or square-root corrections. A second VIF analysis was performed to confirm the reduction in multicollinearity among transformed predictors.

### 3.1. Exploratory Data Analysis

An exploratory assessment of the clinical variables was performed to characterize their distributional behavior and to identify potential issues related to skewness, outliers, and linear dependencies among predictors. This preliminary analysis informed the subsequent steps of variable transformation (Experiment 2) and multicollinearity reduction (both experiments).

#### 3.1.1. Original Histograms of Lipid Profile Variables (Experiment 1)

**Figure 3:**
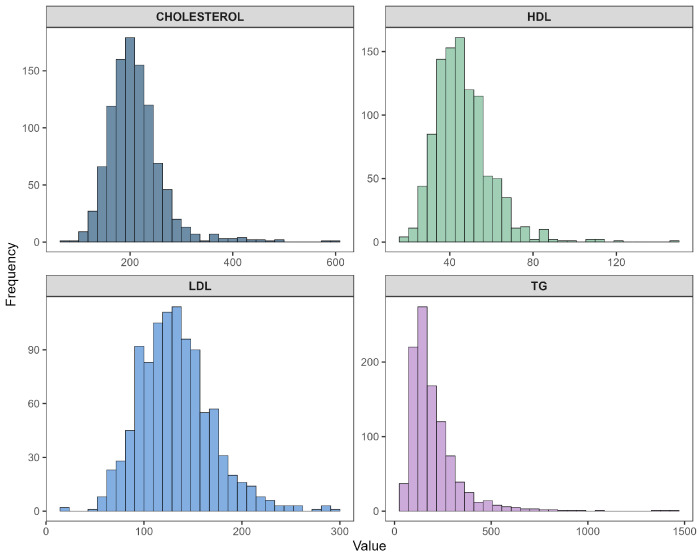
Histograms of original lipid profile variables: total cholesterol, HDL, LDL, and triglycerides.

Figure 3 illustrates the distribution of the four primary lipid biomarkers: total cholesterol, HDL cholesterol, LDL cholesterol, and triglycerides. Cholesterol and LDL displayed moderately right-skewed distributions. HDL exhibited a narrower and nearly symmetric distribution with a slight left tail, whereas triglycerides showed a pronounced right-skew, with a long tail extending toward higher values. Such skewness later justified the need for transformation-based correction in Experiment 2.

#### 3.1.2. Original Histograms of Atherogenic Indices (Experiment 1)

**Figure 4:**
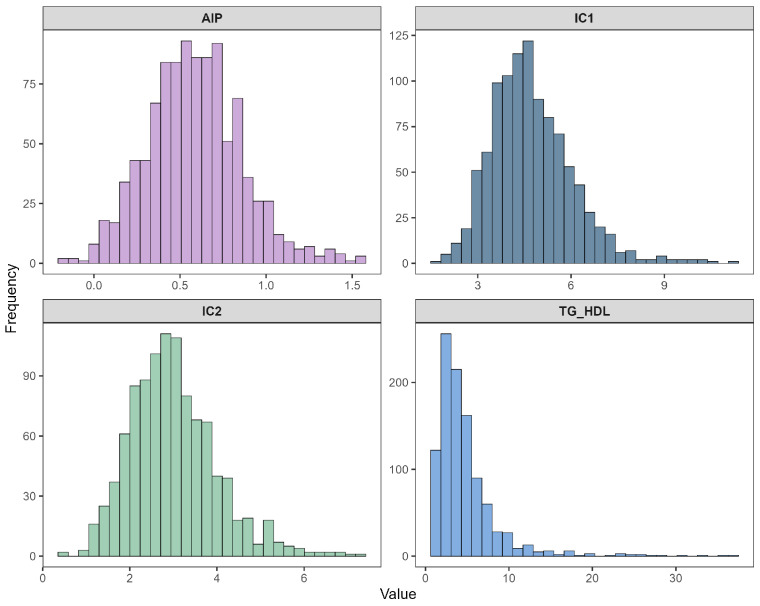
Histograms of atherogenic indices: IC1, IC2, TG/HDL, and AIP.

Figure 4 presents the distributions of the four atherogenic indices derived from the lipid profile: Castelli Index I (IC1), Castelli Index II (IC2), TG/HDL ratio, and the Atherogenic Index of Plasma (AIP). IC1 and IC2 showed moderately skewed distributions, while AIP demonstrated near-symmetry around its central range, reflecting the stabilizing effect of the logarithmic transformation embedded in its definition. In contrast, TG/HDL again displayed severe right-skewness, paralleling the behavior observed in triglycerides. These distributional patterns further reinforced the need for additional transformation strategies to improve normality in Experiment 2.

#### 3.1.3. Correlation Structure of Lipid and Atherogenic Variables (Experiment 1)

**Figure 5:**
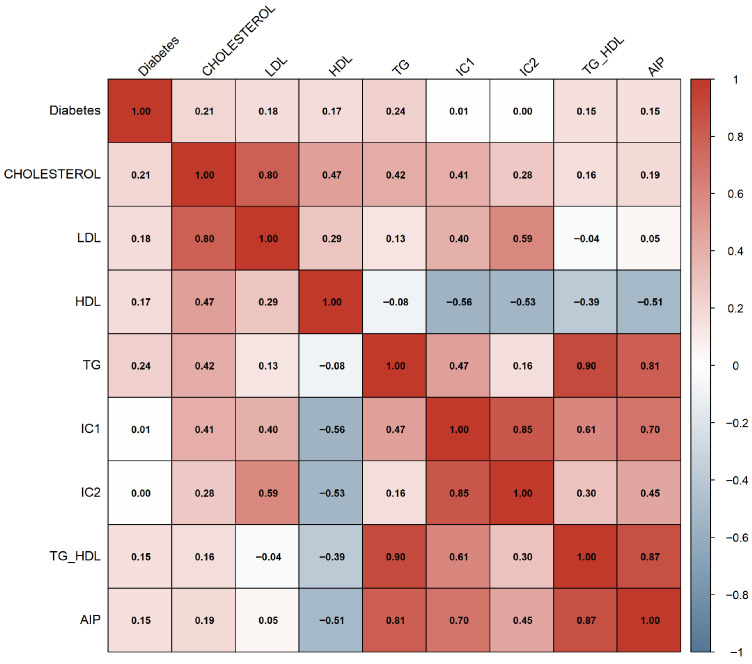
Pearson correlation matrix of lipid variables and atherogenic indices.

Figure 5 displays the Pearson correlation matrix computed for the original lipid biomarkers and the derived atherogenic indices. As expected, strong positive correlations were observed among total cholesterol, LDL, IC1, and IC2, reflecting their shared biochemical origin. HDL showed moderate negative correlations with IC1, IC2, and AIP, consistent with its protective metabolic role. The TG/HDL ratio and AIP exhibited exceptionally strong positive correlations (r>0.80), given that AIP is derived directly from the logarithm of TG/HDL.

These high-magnitude associations suggested substantial redundancy among several lipid-derived features, providing an early indication that multicollinearity could compromise modeling performance. This finding justified the subsequent Variance Inflation Factor (VIF) analysis performed in both experiments to refine the feature set.

### 3.2. Normality Assessment (Experiment 2 Only)

#### 3.2.1. Histogram Analysis Before Transformation

**Figure 6:**
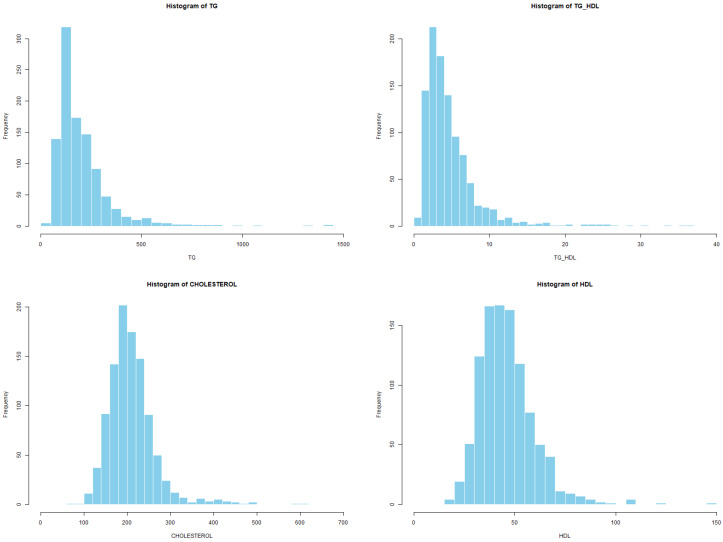
Representative histograms of lipid variables before transformation.

Figure 6 presents representative histograms of key lipid variables before applying any statistical transformations. Triglycerides (TGs) show a strongly right-skewed distribution, with most observations concentrated between approximately 100–300 mg/dL and a long heavy tail extending beyond 1000 mg/dL. A similar pattern is observed for the TG/HDL ratio, which exhibits pronounced positive skewness with values extending above 25.

Total cholesterol displays a moderately right-skewed distribution, with a dense central region around 180–240 mg/dL and a tail toward higher concentrations. In contrast, HDL shows an approximately symmetric distribution with only mild skewness, serving as a reference for a near-normal lipid variable.

To address the observed skewness, logarithmic and square-root transformations were applied as part of Experiment 2.

#### 3.2.2. QQ-Plots Before Transformation

**Figure 7:**
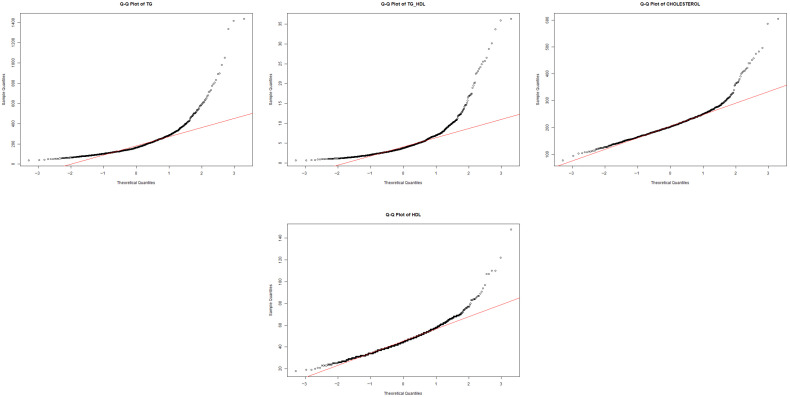
Representative QQ-plots of lipid variables before transformation. Black dots represent the empirical quantiles of the transformed data, while the red line corresponds to the theoretical quantiles of a normal distribution. Deviations from the reference line indicate departures from normality, particularly in the distribution tails.

Figure 7 shows representative QQ-plots for the most skewed lipid variables prior to transformation. The QQ-plot of triglycerides (TGs) exhibits a pronounced upward deviation in the upper quantiles, reflecting heavy right-tailed skewness and clear departure from normality. A similar pattern is observed for the TG/HDL ratio, where extreme values diverge substantially from the theoretical line, confirming the asymmetric long-tail behavior also detected in the corresponding histograms. Even variables with more moderate skewness, such as total cholesterol and HDL, display curvature and deviations at the extremes, indicating that none of the lipid-related variables satisfy the normality assumption in their original scale.

#### 3.2.3. Statistical Tests of Normality

Before applying transformation procedures in Experiment 2, the distributional properties of all continuous variables were evaluated using four complementary statistical tests: Kolmogorov–Smirnov (KS), Shapiro–Wilk (SW), Anderson–Darling (AD), and Jarque–Bera (JB). The combined results, summarized in Table 3, indicate that nearly all variables exhibited statistically significant deviations from normality (p<0.05) across all tests.

Visual inspection through histogram plots and Q–Q plots (Figure 6 and Figure 7) further confirmed non-normal patterns, including pronounced right-tailed skewness in triglycerides (TGs) and TG/HDL, mild-to-moderate skewness in CHOLESTEROL, LDL and IC indices, and moderate asymmetry in anthropometric variables such as WEIGHT, WAIST, HIP and BMI.

**Figure 8:**
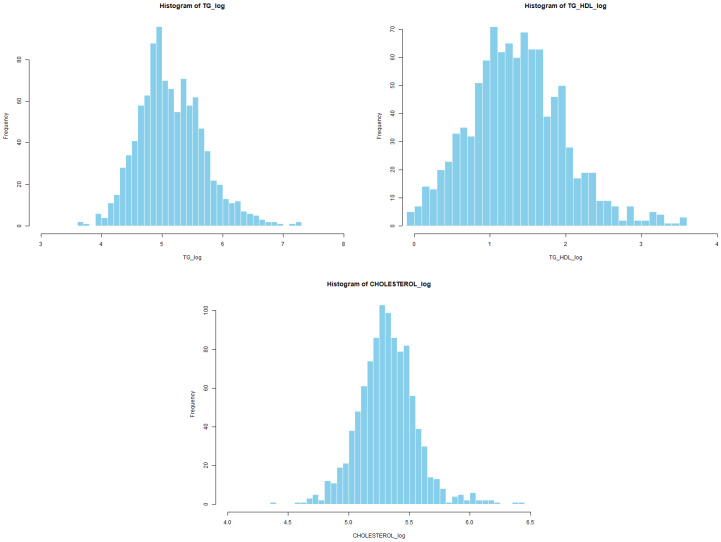
Histograms after applying normality-guided transformations.

Given the consistent evidence of non-normality, variable-specific transformation rules were applied. Logarithmic transformations were assigned to variables with strong right-skewed distributions (CHOLESTEROL, TG, IC1, IC2, TG/HDL), while square-root transformations were used for variables with more moderate skewness (LDL, WEIGHT, WAIST, HIP, BMI). After transformation, new histograms and Q–Q plots (Figure 6 and Figure 8) showed substantially improved alignment with the theoretical normal distribution, supporting the adequacy of the selected transformations.

These improved distributional properties contributed to stabilized variance across predictors, reduced the influence of extreme outliers, and ensured compatibility with downstream methods sensitive to non-normality (e.g., Pearson correlation, VIF analysis).

#### 3.2.4. Histogram Analysis After Transformation

The resulting distributions are shown in Figure 8. The log-transformed TG variable exhibits markedly reduced skewness and improved symmetry, with values concentrated around log(TG) = 4.8–5.4. The TG/HDL ratio also shows substantial correction after log-transformation, producing a more symmetric and compact distribution.

The logarithmic transformation of total cholesterol further reduces asymmetry and yields a distribution that closely approximates normality. HDL was not transformed, as its original distribution already showed acceptable symmetry.

#### 3.2.5. QQ-Plots After Transformation

**Figure 9:**
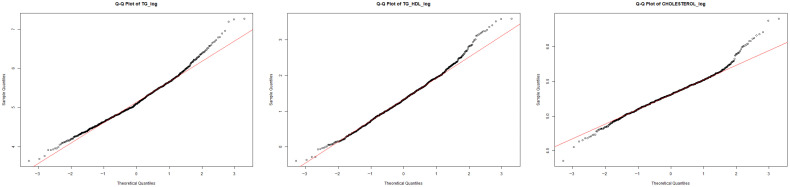
QQ-plots of transformed lipid variables (log-based transformations). Black dots represent the empirical quantiles of the transformed data, while the red line corresponds to the theoretical quantiles of a normal distribution. Deviations from the reference line indicate departures from normality, particularly in the distribution tails.

After applying the variable-specific transformations described in Section 3.2.3, the normality of the transformed lipid variables improved substantially. Figure 9 presents the QQ-plots of TG_log, TG_HDL_log, and CHOLESTEROL_log, which now align much more closely with the theoretical reference line. These visual results indicate a marked reduction in skewness and tail heaviness, particularly for triglycerides and TG/HDL, whose transformed versions exhibit nearly linear patterns. Although slight deviations remain at the extreme quantiles, the transformed distributions approximate normality far better than their raw counterparts, supporting their suitability for Experiment 2 and its modeling procedures.

### 3.3. Multicollinearity Analysis

Multicollinearity was evaluated in both experiments using the Variance Inflation Factor (VIF), a widely used diagnostic to quantify redundancy among predictors in clinical datasets. Features with VIF values greater than 10 were considered highly collinear and therefore unsuitable for inclusion in the final modeling pipeline.

#### 3.3.1. Experiment 1: VIF Results Before Transformation

Table 4 reports the VIF values computed using the original (untransformed) non-glycemic variables. Strong multicollinearity was observed among lipid components and atherogenic indices. In particular, Cholesterol, LDL, IC1, and IC2 exhibited VIF values well above conventional thresholds, reflecting their strong linear dependence. Similarly, the TG/HDL ratio showed substantial redundancy with TG and HDL.

Based on these results, only variables with VIF values below 10 were retained for Experiment 1, namely Age, Gender, Education, Height, Waist, Hip, WHR, Systolic BP, Diastolic BP, and AIP. Accordingly, IC1, IC2, and TG/HDL were excluded from Experiment 1. In contrast, AIP—although derived from TG/HDL—showed a considerably lower VIF than the other indices, supporting its retention as the principal lipid-derived predictor.

#### 3.3.2. Experiment 2: VIF Results After Transformations

After applying the normality-guided transformations to lipid and anthropometric variables, VIF values were recomputed. Table 5 shows that collinearity decreased for several features; however, some transformed variables (e.g., IC2_log, LDL_sqrt, TG_log) still exhibited elevated VIF values due to their strong dependence on the original lipid components.

Following the same criteria as in Experiment 1, only variables with VIF values below 10 were retained for Experiment 2. The final feature set consisted of Age, Gender, Education, Height, WHR, Systolic BP, Diastolic BP, Cholesterol_log, and Waist_sqrt. Transformed features with persistently high VIF values were therefore removed, ensuring acceptable collinearity levels and numerical stability for model training.

### 3.4. Experiment 1: Baseline Classification Results

Experiment 1 evaluated the predictive performance of multiple machine learning algorithms using anthropometric variables, blood pressure measurements, and the Atherogenic Index of Plasma (AIP) as the sole lipid-derived predictor. All continuous features were standardized using Z-score normalization prior to the 70–30 train–test split, ensuring consistent scaling across heterogeneous clinical units. This experiment represents the baseline scenario, where no statistical transformations were applied to lipid variables.

#### 3.4.1. Confusion Matrices and Performance Metrics

Table 6, Table 7, Table 8 and Table 9 summarize the confusion matrices for LR, RF, SVM-RBF, and XGBoost. Derived performance metrics—including accuracy, precision, recall (sensitivity), specificity, and F1-score—are presented in Table 10. These metrics provide a comprehensive evaluation of the classification behavior of each model.

Overall, all models demonstrated strong discriminative ability, with accuracies ranging between 0.83 and 0.85. SVM and RF achieved the highest F1-scores, whereas XGBoost produced the highest sensitivity, indicating superior identification of diabetic individuals.

#### 3.4.2. ROC Curve Analysis in Experiment 1

**Figure 10:**
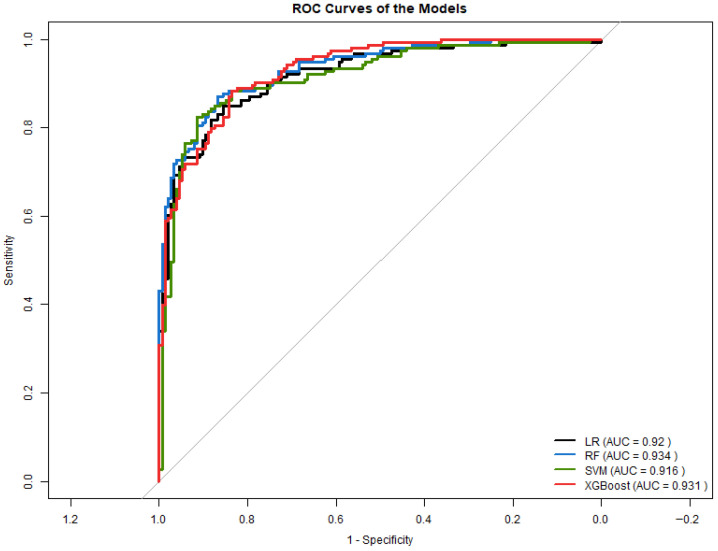
ROC curves for all classification models in Experiment 1.

Figure 10 shows the ROC curves for all evaluated models. Models exhibited strong discrimination, with AUC values ranging from 0.916 to 0.934. Random Forest achieved the highest AUC (0.934), closely followed by XGBoost (0.93). The high AUC scores highlight the robust predictive value of anthropometric and hemodynamic variables combined with AIP.

#### 3.4.3. Summary of Experiment 1

Experiment 1 demonstrates that even without transformed lipid features, AIP remains a strong independent predictor of T2D when combined with anthropometric and blood-pressure variables. Tree-based models (RF and XGBoost) showed slightly superior classification performance, whereas SVM delivered the best balance between sensitivity and specificity. These results establish a solid baseline for evaluating the impact of lipid-transformations introduced in Experiment 2.

### 3.5. Experiment 2: Classification Results with Transformed Lipid Variables

Experiment 2 evaluated the effect of applying statistically guided transformations to lipid variables—specifically logarithmic transformations for variables with strong right skewness (CHOL, TG, IC1, IC2, TG_HDL) and square-root transformations for moderately skewed anthropometric traits (LDL, WEIGHT, WAIST, HIP, BMI)—prior to multicollinearity assessment and model training. These transformations substantially improved distributional symmetry, as verified through histograms and QQ-plots (Figure 8 and Figure 9), and allowed a refined feature selection through VIF analysis (Table 5).

#### 3.5.1. Confusion-Matrix-Based Performance Metrics

Table 11, Table 12, Table 13 and Table 14 summarize the confusion matrices obtained for Logistic Regression (LR), Support Vector Machines with RBF kernel (SVM), XGBoost, and Random Forest (RF). From these matrices, accuracy, precision, recall, specificity, and F1-score were computed and are reported in Table 15.

Overall, SVM exhibited the highest performance across metrics, achieving an accuracy of 0.859, precision of 0.867, and F1-score of 0.858. RF also showed competitive performance with a sensitivity of 0.863. XGBoost, although slightly lower in accuracy (0.823), maintained balanced sensitivity and specificity.

#### 3.5.2. ROC Curve Analysis in Experiment 2

**Figure 11:**
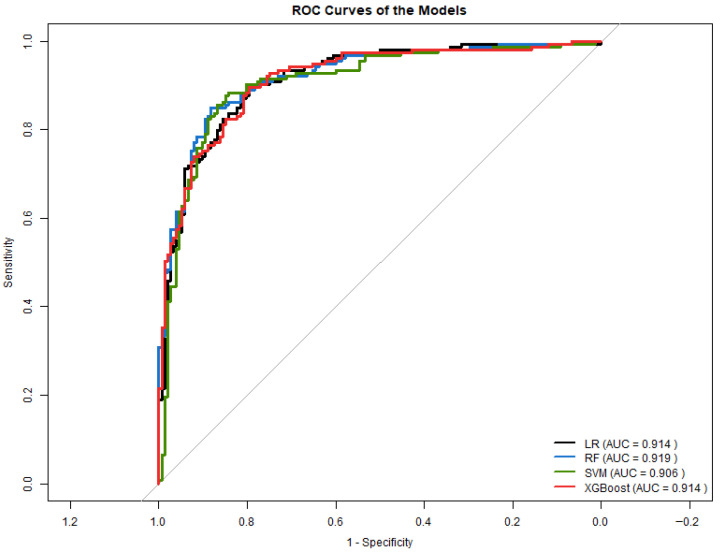
ROC curves for all models in Experiment 2.

Figure 11 displays the ROC curves for all models. SVM and Random Forest achieved the highest AUC values (0.906 and 0.919, respectively), while LR and XGBoost obtained AUCs of 0.914.

#### 3.5.3. Comparison with Experiment 1

Compared to Experiment 1, Experiment 2 produced:

- Higher overall accuracy for SVM and RF.
- Improved class separation, reflected in ROC curves.
- Better precision and F1-score in SVM due to reduced skewness and stabilized variance.
- More balanced sensitivity and specificity across models.

These findings suggest that incorporating normality-guided transformations enhanced model performance, particularly for algorithms sensitive to feature scaling and distribution such as SVM and LR. Although ensemble models (RF, XGBoost) showed moderate improvements, their gains were less pronounced.

Overall, Experiment 2 demonstrates that correcting heavy-tailed lipid distributions and reducing multicollinearity yields generalizable models for predicting type 2 diabetes from non-glycemic clinical variables.

## 4. Discussion

The present work investigated whether routinely available non-glycemic clinical variables—primarily lipid profile components and anthropometric measures—can support early detection of T2D through ML. The findings of both experiments provide evidence that lipid-derived indices, when adequately preprocessed and combined with anthropometric parameters, offer meaningful predictive information for identifying metabolic dysregulation even in the absence of classical glycemic biomarkers.

### 4.1. Interpretation of Main Findings

Across both experiments, all machine learning models demonstrated moderate to high discriminative capacity, with AUC values ranging from 0.90 to 0.93. These values are comparable to those reported in recent literature investigating non-glycemic predictors of dysglycemia. In Experiment 1, AIP was the only lipid-derived variable retained due to multicollinearity constraints. Even in this conservative setting, the models achieved competitive performance, supporting prior evidence that AIP reflects underlying insulin resistance and cardiometabolic risk.

Experiment 2 incorporated a broader set of lipid variables after resolving skewness and multicollinearity through log and square-root transformations. These transformations improved distributional symmetry, reduced VIF values, and consequently enabled the inclusion of CHOLESTEROL_log, TG_log, and WAIST_sqrt in model training. The performance improvements observed—particularly in SVM and Random Forest—indicate that the preservation of lipid variability contributes additional information relevant to early metabolic impairment.

The enhanced performance in Experiment 2 aligns with the well-established nonlinear nature of lipid metabolism in the progression toward dysglycemia. Several studies have documented nonlinear and threshold effects between atherogenic indices and T2D risk [13], reinforcing the rationale for applying transformations that stabilize variance and reduce tail influence.

### 4.2. Comparison with Previous Studies

Recent research has increasingly explored the application of machine learning techniques for the identification of type 2 diabetes and related metabolic conditions using heterogeneous clinical, anthropometric, lifestyle, and alternative data sources. In a large-scale electronic health record study, Luo et al. [29] employed an explainable XGBoost model to stratify individuals at the onset of prediabetes, achieving an AUC of 0.816. Their framework incorporated glycemic variables, including glycated hemoglobin (HbA1c), together with non-glycemic clinical features such as body mass index, blood pressure, lipid profiles, liver enzymes, medication use, and lifestyle factors. Despite the inclusion of glycemic information, their results highlighted the substantial contribution of non-glycemic variables to early risk stratification.

The exploration of non-traditional and non-invasive data modalities has also gained attention. Oreskovic et al. [30] proposed voice-based machine learning models combined with basic demographic and anthropometric variables to predict prediabetes across geographically distinct populations. Notably, their models did not include direct glycemic measurements, relying instead on voice-derived features, body mass index, age, and sex. Their results showed moderate balanced accuracy, with notable differences between female and male subgroups, emphasizing challenges related to model generalization and population heterogeneity.

Several studies have focused on comparative evaluations of classical and ensemble machine learning algorithms using routine health examination data. Deberneh and Kim [31] conducted a comprehensive comparison of logistic regression, Random Forest, support vector machines, XGBoost, and ensemble models for type 2 diabetes prediction. Their models incorporated both glycemic variables, including fasting plasma glucose and glycated hemoglobin, and non-glycemic predictors such as anthropometric measurements, lipid-related parameters, and lifestyle factors, reporting competitive classification metrics across algorithms. Similarly, Almadhoun and Burhanuddin [32] demonstrated that optimized feature selection strategies substantially improve predictive performance for early detection of prediabetes risk; however, fasting blood glucose remained part of their feature set, indicating that glycemic information contributed to model performance.

From a methodological perspective, Abousaber et al. [33] introduced a robust predictive framework designed to address class imbalance in diabetes classification tasks. Their comparative analysis included multiple public datasets and a wide range of machine learning algorithms, several of which incorporated glycemic variables such as plasma glucose as part of the input features, alongside non-glycemic clinical parameters. The study confirmed the consistent superiority of ensemble and tree-based approaches across multiple performance metrics, including ROC-AUC.

Other investigations have highlighted the relevance of lifestyle and population-specific factors. Sun et al. [34] applied machine learning techniques to identify dietary patterns associated with obesity-related type 2 diabetes risk in elderly Chinese men. Their models integrated metabolic factors and anthropometric variables, including glycemic measurements such as fasting blood glucose, in combination with dietary and lifestyle information, demonstrating that interactions between these domains play a significant role in disease risk. In contrast, Ge et al. [35] examined the association between body mass index and cognitive impairment among individuals with established type 2 diabetes using regression-based models. This study did not focus on diabetes detection and did not employ glycemic variables as predictors, as diabetes status was predefined; instead, it illustrated the complex and nonlinear influence of anthropometric indicators on diabetes-related outcomes.

More recently, Kimura et al. [36] proposed digital bioimpedance measurements as non-invasive biomarkers for physical activity detection in individuals with type 2 diabetes. Their logistic regression models incorporated baseline glycated hemoglobin (HbA1c) as a reference predictor and evaluated the added value of bioimpedance-derived features, achieving an AUC of 0.847. This work reflects an emerging trend toward integrating physiological signals with both glycemic and non-glycemic clinical variables to enhance diabetes-related risk assessment.

In comparison with these studies, the present work demonstrates that competitive and, in several cases, superior discriminative performance can be achieved using exclusively non-glycemic variables when multicollinearity is rigorously controlled and lipid-related markers are appropriately engineered. As summarized in Table 16, prior investigations typically report AUC values in the range of approximately 0.80 to 0.85, whereas the models developed in this study achieved AUC values between 0.91 and 0.93 using only non-glycemic clinical variables. These findings suggest that the systematic incorporation of lipid-based markers and atherogenic indices, combined with strict feature selection and transformation strategies, substantially enhances the predictive accuracy of non-glycemic type 2 diabetes risk modeling.

### 4.3. Clinical Implications

The results highlight that lipid biomarkers—routinely measured, and obtainable without fasting—contain latent metabolic information useful for early T2D screening. This is clinically relevant given that:

- many patients undergo annual lipid panels but not glycemic testing;
- fasting requirements limit adherence to HbA1c or OGTT screening;
- primary-care settings prioritize inexpensive and easily accessible biomarkers.

Our findings support the possibility of implementing ML-based pre-screening tools using existing electronic health record (EHR) data to flag individuals at risk before glycemic abnormalities become clinically evident.

### 4.4. Methodological Considerations

This study emphasizes the importance of preprocessing in biomedical ML. Experiment 1 showed that multicollinearity among lipid variables—particularly TG, LDL, IC1, and IC2—can distort model coefficients and degrade linear models. Experiment 2 demonstrated that appropriate transformations not only mitigate skewness but also reduce collinearity, allowing models to incorporate richer lipid information.

The improved performance observed in SVM and LR after transformation underscores the sensitivity of margin-based and linear classifiers.

## 5. Conclusions

This study evaluated whether routinely collected non-glycemic clinical variables—particularly lipid profile components, atherogenic indices, and anthropometric measures—can support the early detection of T2D using machine learning (ML). Across two complementary experiments, the evaluated models achieved high discriminative performance, with AUC values ranging from 0.90 to 0.93. In Experiment 1, the best-performing models yielded AUC values between 0.90 and 0.92, while in Experiment 2, AUC values increased slightly, reaching up to 0.93 after distribution-aware preprocessing. Importantly, model performance was not assessed solely through AUC; confusion matrix analysis showed false positive counts ranging from 17 to 23 in Experiment 1 and from 21 to 27 in Experiment 2, depending on the classifier. These results indicate that lipid-derived information alone can effectively distinguish diabetic from non-diabetic adults with clinically relevant accuracy while maintaining an acceptable false positive burden.

### 5.1. Conclusions from Experiment 1

Experiment 1 focused on evaluating a minimal diagnostic feature set composed of anthropometric measures, blood pressure, and the Atherogenic Index of Plasma (AIP), after explicitly controlling for multicollinearity. The consistent performance observed across all evaluated classifiers confirms that AIP alone captures relevant metabolic alterations associated with T2D. These results demonstrate that simplified, non-glycemic feature sets can retain meaningful diagnostic value in early screening contexts.

### 5.2. Conclusions from Experiment 2

Experiment 2 extended the analysis by incorporating additional lipid variables following normality-guided transformations and VIF-based feature selection. This strategy enabled the use of a richer lipid representation and improved the stability of model behavior, leading to more balanced sensitivity–specificity profiles, particularly for SVM and Random Forest models. These findings highlight the role of principled preprocessing in enhancing model robustness while preserving interpretability.

### 5.3. General Conclusions

In summary:

- Non-glycemic clinical variables—especially atherogenic indices—contain sufficient diagnostic information to identify individuals with T2D.
- Appropriate preprocessing (distributional transformations and VIF-based feature selection) is essential to stabilize ML models and control FP rates.
- Beyond high AUC values, the explicit evaluation of FP values and specificity provides a more clinically meaningful assessment of model utility.
- Given their low cost, routine availability, and acceptable FP burden, lipid-based markers represent a practical and scalable option for early T2D screening, particularly in resource-limited settings.

### 5.4. Future Work

Future research should prioritize the external validation of the proposed models in independent and geographically diverse cohorts in order to assess their generalizability beyond the population analyzed in this study. In addition, the inclusion of lifestyle-related variables, inflammatory biomarkers, and other routinely collected clinical parameters may further enhance predictive performance and model robustness. Methodologically, future work should also incorporate k-fold cross-validation strategies to provide more robust estimates of model generalization and reduce dependence on a single train–test split. Longitudinal analyses incorporating follow-up data would allow evaluation of these approaches for predicting incident T2D over time, rather than focusing solely on cross-sectional classification. Finally, future studies should explore the integration of these models into clinical decision-support systems and electronic health record platforms, with particular emphasis on interpretability, usability, and feasibility in real-world healthcare settings. In this context, nationally representative datasets such as the ENSANUT survey could be leveraged to evaluate the routine availability of lipid profiles across healthcare institutions in Mexico and to assess the potential population-level applicability of the proposed approach.

## Figures and Tables

**Table 1:** Features and their description in the clinical database.

Feature	Description
ID	Numeric patient identifier.
Age	Patient age (years).
Gender	Biological sex (Male = 0, Female = 1).
Education	Education level.
Weight	Body weight (kg).
Height	Body height (cm).
Waist	Waist circumference (cm).
Hip	Hip circumference (cm).
BMI	Body mass index (kg/m^2^).
WHR	Waist-to-hip ratio.
Systolic BP	Systolic blood pressure (mmHg).
Diastolic BP	Diastolic blood pressure (mmHg).
Cholesterol	Total cholesterol (mg/dL).
LDL	Low-density lipoprotein cholesterol (mg/dL).
HDL	High-density lipoprotein cholesterol (mg/dL).
TGs	Triglycerides (mg/dL).
Glucose	Blood glucose (mg/dL).
MmolGluc	Blood glucose (mmol/L).
Insulin	Serum insulin (μU/mL).
HOMA_IR	Homeostatic model assessment of insulin resistance.
Diabetes	Patient condition (Non-diabetic = 0, Diabetic = 1).

**Table 2:** Structure of the confusion matrix used to compute performance metrics.

Actual/Predicted	Positive (1)	Negative (0)
**Positive (1)**	True Positive (TP)	False Negative (FN)
**Negative (0)**	False Positive (FP)	True Negative (TN)

**Table 3:** Normality test results for all continuous variables in Experiment 2. KS = Kolmogorov–Smirnov, SW = Shapiro–Wilk, AD = Anderson–Darling. “Normal” indicates whether the distribution met normality assumptions (p>0.05).

Variable	KS (*p*)	SW (*p*)	AD (*p*)	Normal
Age	1.12 $\times \;10^{-07}$	7.97 $\times \;10^{-17}$	3.7 $\times \;10^{-24}$	No
WEIGHT	0.0717	1.72 $\times \;10^{-11}$	1.41 $\times \;10^{-06}$	No
HEIGHT	0.0169	3.55 $\times \;10^{-29}$	5.87 $\times \;10^{-10}$	No
WAIST	1.48 $\times \;10^{-06}$	2.12 $\times \;10^{-23}$	3.7 $\times \;10^{-24}$	No
HIP	7.88 $\times \;10^{-08}$	2.45 $\times \;10^{-28}$	3.7 $\times \;10^{-24}$	No
BMI	0.00028	2.82 $\times \;10^{-20}$	2.00 $\times \;10^{-23}$	No
WHR	1.87 $\times \;10^{-38}$	3.35 $\times \;10^{-50}$	3.7 $\times \;10^{-24}$	No
Systolic.BP	6.38 $\times \;10^{-36}$	2.19 $\times \;10^{-16}$	3.7 $\times \;10^{-24}$	No
Diastolic.BP	2.49 $\times \;10^{-34}$	5.66 $\times \;10^{-19}$	3.7 $\times \;10^{-24}$	No
CHOLESTEROL	2.36 $\times \;10^{-09}$	1.67 $\times \;10^{-27}$	3.7 $\times \;10^{-24}$	No
LDL	0.0260	4.00 $\times \;10^{-12}$	1.47 $\times \;10^{-10}$	No
HDL	1.49 $\times \;10^{-04}$	1.90 $\times \;10^{-22}$	2.2 $\times \;10^{-24}$	No
TG	8.03 $\times \;10^{-20}$	2.31 $\times \;10^{-38}$	3.7 $\times \;10^{-24}$	No
IC1	0.00054	7.81 $\times \;10^{-18}$	1.87 $\times \;10^{-19}$	No
IC2	0.00051	4.16 $\times \;10^{-17}$	3.2 $\times \;10^{-24}$	No
TG_HDL	9.52 $\times \;10^{-02}$	9.38 $\times \;10^{-40}$	2.49 $\times \;10^{-15}$	No
AIP	0.33417	6.44 $\times \;10^{-06}$	0.0014	No

**Table 4:** VIF evaluation of clinical and lipid variables (Experiment 1).

Feature	VIF
Age	1.37
Gender	2.66
Cholesterol	65.79
LDL	45.69
HDL	12.56
TG	25.41
Education	1.33
Weight	12.70
Height	6.13
Waist	7.94
Hip	8.72
BMI	10.85
WHR	3.80
Systolic BP	1.80
Diastolic BP	1.70
IC1	80.44
IC2	63.60
TG/HDL	26.72
AIP	7.60

**Table 5:** VIF evaluation after normality-guided transformations (Experiment 2).

Feature	VIF
Age	1.37
Gender	2.65
Education	1.34
Height	8.45
WHR	4.35
Systolic BP	1.78
Diastolic BP	1.71
HDL	19.21
AIP	278.33
IC2_log	63.97
LDL_sqrt	53.66
Weight_sqrt	17.65
Waist_sqrt	8.59
Hip_sqrt	10.77
BMI_sqrt	15.59
Cholesterol_log	5.66
TG_log	207.12

**Table 6:** Confusion matrix for LR in Experiment 1.

Prediction/True	Class 0	Class 1
Class 0	124	23
Class 1	28	130

**Table 7:** Confusion matrix for Random Forest in Experiment 1.

Prediction/True	Class 0	Class 1
Class 0	123	18
Class 1	29	135

**Table 8:** Confusion matrix for SVM (RBF) in Experiment 1.

Prediction/True	Class 0	Class 1
Class 0	129	22
Class 1	23	131

**Table 9:** Confusion matrix for XGBoost in Experiment 1.

Prediction/True	Class 0	Class 1
Class 0	121	17
Class 1	31	136

**Table 10:** Comparison of performance metrics for all models in Experiment 1.

Model	Accuracy	Precision	Recall	Specificity	F1-Score
LR	0.833	0.823	0.850	0.816	0.836
RF	0.846	0.823	0.882	0.809	0.852
SVM (RBF)	0.852	0.851	0.856	0.849	0.853
XGBoost	0.843	0.814	0.889	0.796	0.850

**Table 11:** Confusion matrix for Logistic Regression in Experiment 2.

Prediction/Actual	Class 0	Class 1
Class 0	129	27
Class 1	23	126

**Table 12:** Confusion matrix for SVM (RBF) in Experiment 2.

Prediction/Actual	Class 0	Class 1
Class 0	132	23
Class 1	20	130

**Table 13:** Confusion matrix for XGBoost in Experiment 2.

Prediction/Actual	Class 0	Class 1
Class 0	124	26
Class 1	28	127

**Table 14:** Confusion matrix for Random Forest in Experiment 2.

Prediction/Actual	Class 0	Class 1
Class 0	125	21
Class 1	27	132

**Table 15:** Comparison of performance metrics in Experiment 2.

Model	Accuracy	Precision	Recall	Specificity	F1-Score
LR	0.836	0.846	0.824	0.849	0.834
RF	0.843	0.830	0.863	0.822	0.846
SVM	0.859	0.867	0.850	0.868	0.858
XGBoost	0.823	0.819	0.830	0.816	0.825

**Table 16:** Comparison of predictive performance between previous studies and the present work.

Study	Model	Reported Performance Metrics
Luo et al. (2025) [29]	XGBoost	ROC-AUC = 0.816
Oreskovic et al. (2025) [30]	RF/XGBoost	Balanced accuracy = 0.78 (female), 0.68 (male)
Deberneh & Kim (2021) [31]	LR/RF/SVM/XGBoost/Ensemble	Accuracy = 0.71–0.73; Precision = 0.71–0.74; Recall = 0.71–0.74; F1-score = 0.71–0.74
Almadhoun & Burhanuddin (2025) [32]	Random Forest	Cross-validated ROC-AUC = 0.911
Sun et al. (2025) [34]	12 Machine learning models	Classification accuracy ≈ 0.73–0.75
Ge et al. (2025) [35]	Logistic regression	Association analysis (no classification AUC reported)
Kimura et al. (2025) [36]	Logistic regression	ROC-AUC = 0.847
Present work	Random Forest	Accuracy = 0.84; Sensitivity = 0.83; Specificity = 0.82; ROC-AUC = 0.91–0.93

## Data Availability

The original contributions presented in this study are included in the article. Further inquiries can be directed to the corresponding authors.

## Abbreviations

The following abbreviations are used in this manuscript:

AD	Anderson–Darling test
AIP	Atherogenic Index of Plasma
AUC	Area Under the (ROC) Curve
BMI	Body Mass Index
BP	Blood Pressure
CVD	Cardiovascular Disease
ENSANUT	Encuesta Nacional de Salud y Nutrición (Mexican National Health and Nutrition Survey)
EHR	Electronic Health Record
HbA1c	Glycated Hemoglobin
HDL	High-Density Lipoprotein Cholesterol
HOMA-IR	Homeostatic Model Assessment of Insulin Resistance
IC1	Castelli Risk Index I (Total Cholesterol/HDL)
IC2	Castelli Risk Index II (LDL/HDL)
IDF	International Diabetes Federation
IMSS	Instituto Mexicano del Seguro Social
JB	Jarque–Bera test
KS	Kolmogorov–Smirnov test
LDL	Low-Density Lipoprotein Cholesterol
LR	Logistic Regression
ML	Machine Learning
OGTT	Oral Glucose Tolerance Test
RF	Random Forest
RBF	Radial Basis Function
ROC	Receiver Operating Characteristic
SVM	Support Vector Machine
SVM-RBF	Support Vector Machine with Radial Basis Function kernel
T2D	Type 2 Diabetes
TGs	Triglycerides
TG/HDL	Triglyceride-to-HDL Cholesterol Ratio
VIF	Variance Inflation Factor
WHR	Waist-to-Hip Ratio
XGBoost	Extreme Gradient Boosting
Z-score	Standard Score Normalization
